# A Case of Suspected Multiple Acyl‐CoA Dehydrogenase Deficiency‐Induced Encephalopathy

**DOI:** 10.1155/crcc/9093349

**Published:** 2026-08-19

**Authors:** Molly Hirsh, Lauren McCroskie, Julia Weaver, Kathryn Humes, Nicola M. Zetola

**Affiliations:** ^1^ Augusta University/University of Georgia Medical Partnership, Athens, Georgia, USA; ^2^ Dell Medical School–University of Texas at Austin, Austin, Texas, USA; ^3^ Medical College of Georgia, Augusta, Georgia, USA, gru.edu; ^4^ The Ohio State University Wexner Medical Center, Columbus, Ohio, USA, osu.edu

**Keywords:** electron transfer flavoprotein dehydrogenase, glutaric aciduria Type II, hyperammonemia, lipid storage myopathy, MADD, metabolic disorder, multiple acyl-coenzyme A dehydrogenase deficiency

## Abstract

Multiple acyl‐coenzyme A dehydrogenase deficiency (MADD) is a rare inherited disorder that disrupts fatty acid metabolism. We report a case of a patient who presented with confusion, undifferentiated shock, and rapidly worsening lactic acidosis and hyperammonemia, unexplained in severity by primary liver dysfunction. Metabolic investigations suggested the probable cause was late‐onset MADD, likely triggered by pneumonia and exacerbated by early administration of fatty acid‐containing sedatives. Early recognition of MADD and other metabolic disorders, whether inherited or acquired, is crucial for timely diagnosis and management. Unexplained hyperammonemia and other metabolic abnormalities should prompt clinicians to consider these rare conditions.

## 1. Introduction

Multiple acyl‐coenzyme A dehydrogenase deficiency (MADD), also known as glutaric aciduria Type II, is a rare autosomal recessive disorder affecting fatty acid metabolism [1]. It results from defects in electron transfer flavoprotein (ETF) or electron transfer flavoprotein dehydrogenase (ETFDH), enzymes essential for fatty acid *β*‐oxidation within the mitochondrial matrix. In late‐onset presentations (Type III), ETFDH mutations are more common. MADD occurs at an estimated frequency of 1 in 250,000, with adult‐onset Type III being the most prevalent.

Clinical manifestations of MADD include muscle weakness, exercise intolerance, muscle pain, and metabolic decompensation—often associated with episodes of rhabdomyolysis. These manifestations are typically precipitated by metabolic stressors such as fasting or infection. Cardiac arrhythmias, diastolic heart failure, episodic vomiting, hypoglycemia, metabolic acidosis, sensory neuropathy, proximal myopathy, and respiratory failure have also been reported as precipitating factors [1].

There are no formal diagnostic criteria for MADD, but findings may include elevated serum acylcarnitine and increased urinary organic acids. Nonspecific findings can include nonketotic hypoglycemia, metabolic acidosis, hyperammonemia, elevated liver transaminases, and raised creatine kinase. Genetic testing, particularly for ETFDH mutations, may confirm the diagnosis [1].

Management strategies include dietary modifications (low protein and fat), avoidance of fasting, high‐dose riboflavin supplementation, and carnitine replacement in carnitine‐deficient patients. Coenzyme Q10 may also be helpful. Inpatient management often requires intravenous dextrose and bicarbonate therapy, tailored to the patient′s metabolic status [1].

## 2. Objective

We present the case of a woman with confusion, undifferentiated shock, and rapidly worsening hyperammonemia, ultimately suspected to have Type III MADD. We explore the diagnostic challenge of this rare disorder and its impact on potentially fatal patient outcomes. This case highlights the clinical signs of MADD, triggers of metabolic decompensation, and emphasizes the importance of timely consideration and management of MADD.

## 3. Case Report

A 51‐year‐old woman with Bipolar I disorder presented from a local county prison to an outside facility with altered mental status, confusion, abnormal speech, and ventricular tachycardia following 10 days of nausea and vomiting. Pertinent lab results there revealed normal renal and hepatic function, lactic acid of 9.2 mmol/L, creatine kinase of 396 U/L, troponin of 32 ng/mL, but no significant ammonia elevation or evidence of infection. Imaging studies, including a chest x‐ray and CT of the head were unremarkable. A CT chest, abdomen, and pelvis showed diffuse hepatic steatosis and no focal lesions with no other acute processes. In the setting of extreme agitation, the patient received haloperidol, diphenhydramine, and ziprasidone. Her ventricular tachycardia was treated with diltiazem. She was subsequently intubated for airway protection and sedated with fentanyl and propofol. At this time, she was treated with intravenous lactated ringers, empiric vancomycin, and norepinephrine 0.4 mg. She was transferred to a larger facility for further investigation of undifferentiated shock.

Upon arrival, the patient underwent a thorough workup for undifferentiated shock and encephalopathy. Lumbar puncture, blood cultures, and urine cultures were negative. Her urine drug screen was negative. Valproic acid and salicylate levels were within normal limits. Electroencephalogram showed diffuse encephalopathy with no evidence of seizures. Repeat chest imaging revealed multifocal pneumonia. A subsequent bronchoalveolar lavage revealed methicillin‐resistant *Staphylococcus aureus* susceptible to vancomycin.

**Figure 1:**
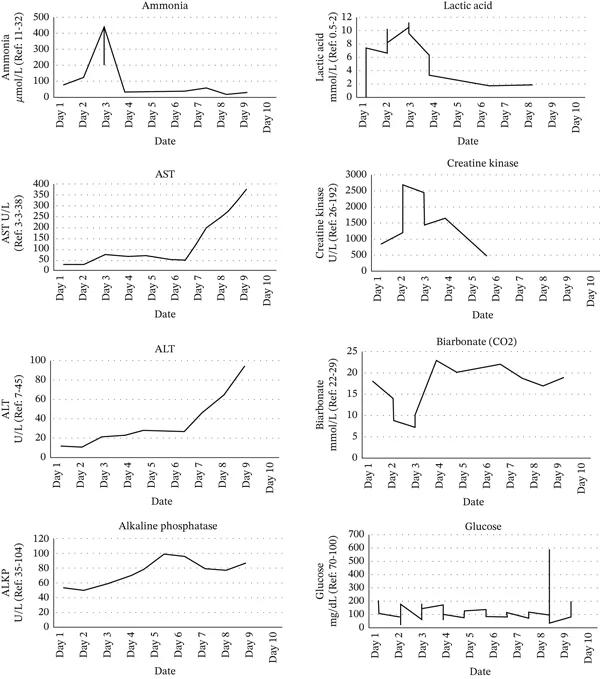
Key case laboratory trends. Legend: Laboratory results over time of patient presenting with lactic acidosis and rising levels of ammonia in a case of suspected multiple acyl CoA dehydrogenase deficiency.

Key patient laboratory results and trends are shown in Table 1 and Figure 1. Despite continued appropriate antibiotic coverage and norepinephrine support, the patient′s lactic acid continued to rise, peaking at 11.2 mmol/L. Her creatine kinase levels peaked at 2706 U/L around the same date. The patient also developed profound hypoglycemia with blood sugars in the 20s mg/dL, and persistent metabolic acidosis, prompting initiation of intravenous bicarbonate. Liver function tests were initially normal (AST 30 U/ L, ALT 12 U/L, total bilirubin 0.4 mg/dL); however, her ammonia rose from 77 to 439 umol/L without significant change in aminotransferases. Further evaluation showed that the patient had a coagulopathy with isolated elevation of prothrombin time and international normalized ratio. Fibrinogen was measured at 178 mg/dL, and factor VIII was 446 IU. However, she had a normal partial thromboplastin time of 32 s, suggesting a vitamin K deficiency over primary liver dysfunction. She was given unit of fresh frozen plasma and vitamin K. For her hyperammonemia, she was initiated on lactulose and rifaximin treatment. Given an inadequate response, she was started on CRRT to help clear the ammonia (Table 2). Despite subsequent ammonia clearance and trialed sedation holidays, her mentation did not improve.

**Table 1:** Summary of patient lab results by date.

Date	Lactic acid	Ammonia	CK	Glucose	Bicarb	AST	ALT	ALKP	PT	INR	aPTT
Reference range	0.5–2 mmol/L	11–32 *μ*mol/L	26–192 U/L	70–100 mg/dL	22–29 mEq/L	3–38 U/L	7–45 U/L	35–104 U/L	10.3–13.3 s	0.9–1.1	25–38 s
Day 1	7.4	77	860	110–201	18	30	12	53	21.4	1.8	21.1
Day 2	6.6–10.2	124	1207–2706	25–175	9–14	33	11	50	—	—	—
Day 3	9.7–11.2	201–439	1432–2460	56–183	7–41	76	21	58	63.3	5.4	32.6
Day 4	3.4–6.3	32	1631	56–172	23	67	22	66	20.5	1.8	24.9
Day 5	—	34	—	78–128	20	73	28	78	12.1	1.1	—
Day 6	—	32	464	88–138	21	57	27	100	10.8	1	—
Day 7	1.8	38	—	80–117	22	52	26	95	—	—	—
Day 8	—	56	—	73–119	19	198	47	79	11.9	1	24.2
Day 9	1.9	18	—	37–587	17	264	65	77	—	—	—
Day 10	—	28	—	80–201	19	379	94	87	—	—	—

**Table 2:** Summary of key patient lab results and interventions by date.

Date	Marker	Peak/nadir	Key intervention
Day 1	—	Admission baseline	—
Day 2	CK (U/L)	Peak: 2706	Dextrose 5% in water started
Day 3	Lactic acid (mmol/L)	Peak: 11.2	Propofol started, given one dose of levocarnitine
Day 3	Ammonia (*μ*mol/L)	Peak: 439	
Day 3	Bicarb (mEq/L)	Peak: 41	
Day 4	—	Lactic acid, ammonia improving	CRRT started, regular tube feeds started
Day 5	—	—	Nutrition consult, transition to low protein tube feeds
Day 6	ALKP (U/L)	Peak: 100	—
Day 7	Lactic acid (mmol/L)	Normalized: 1.8	Propofol stopped
Day 8	Glucose (mg/dL)	Peak: 587	Levocarnitine and CoQ10 started
Day 8	Ammonia (*μ*mol/L)	Normalized: 18	—
Day 9	AST (U/L)	Peak: 379	Riboflavin started
Day 9	ALT (U/L)	Peak: 94	—
Day 10	—	—	CRRT stopped

Given her deteriorating metabolic profile and lack of response to standard treatment, other etiologies were investigated for hyperammonemia disproportionate to liver dysfunction, lactic acidosis, and elevated CK. The patient′s family denied known family history including genetic disorders or sudden infant death. A pediatric geneticist and metabolic hotline were contacted for guidance who recommended testing urine organic acids, purines, and pyrimidines; due to the patient′s need for continuous renal replacement treatment, these studies could not be obtained. A specialty Mayo Clinic Laboratories biochemical genetics profile panel was run, including plasma acylcarnitine profiling tandem mass spectrometry. The results revealed abnormalities suggestive of MADD, including multiple elevated acylcarnitine species (Table 3). Additionally, results noted a disturbed carnitine profile with reduced total and free carnitine levels, a relatively abundant esterified fraction and elevated AC/FC ratio of 1.8 (Table 4). In response to these findings, the patient was treated with intravenous levocarnitine, TPN with cyclinex 2 formula (high carb, low protein), as well as the addition of riboflavin 400 mg, carnitine 100 mg/kg/day, and CoQ10 100 mg daily. The plan was for the patient to follow up with the geneticist outpatient for ETFDH testing. Despite treatment, the patient′s condition worsened; after a family discussion, her care was transitioned to comfort measures. She died on hospital Day 10.

**Table 3:** Results of Mayo Clinic Laboratories biochemical genetics profile collected 6/27/2024 03:43 EDT.

Analyte	Normal range	Result	Units	Specimen	Category
Tyrosine	36–113	155	nmol/mL	Serum	Amino acid
Tetradecenoylcarnitine (C14:1)	< 0.24	0.82	nmol/mL	Serum	Long‐chain acylcarnitine
Tetradecanoylcarnitine (C14)	< 0.12	0.57	nmol/mL	Serum	Long‐chain acylcarnitine
Tetradecadienoylcarnitine (C14:2)	< 0.18	0.26	nmol/mL	Serum	Long‐chain acylcarnitine
Stearoylcarnitine (C18)	< 0.14	0.37	nmol/mL	Serum	Long‐chain acylcarnitine
Sarcosine	< 0.20	25	nmol/mL	Serum	Amino acid
Proline	107–383	530	nmol/mL	Serum	Amino acid
Phenylalanine	45–106	113	mol/mL	Serum	Amino acid
Oleylcarnitine (C18:1)	< 0.39	0.57	nmol/mL	Serum	Long‐chain acylcarnitine
Octanedioylcarnitine (C8‐DC)	< 0.19	0.35	nmol/mL	Serum	Medium‐chain acylcarnitine
Methylmalonyl‐/succinylcarnitine (C4‐DC)	< 0.05	0.05	nmol/mL	Serum	Short‐chain acylcarnitine
Lysine	105–335	605	nmol/mL	Serum	Amino acid
Isovaleryl‐/methylbutyrylcarnitine (C5)	< 0.51	2.4	nmol/mL	Serum	Short‐chain acylcarnitine
Isobutyrylcarnitine (C4)	< 0.83	2.91	nmol/mL	Serum	Short‐chain acylcarnitine
Hexanoylcarnitine (C6)	< 0.17	0.26	nmol/mL	Serum	Medium‐chain acylcarnitine
Hexadecenoylcarnitine (C16:1)	< 0.10	0.38	nmol/mL	Serum	Long‐chain acylcarnitine
Hexadecanoylcarnitine (C16)	< 0.23	0.74	nmol/mL	Serum	Long‐chain acylcarnitine
Glutarylcarnitine (C5‐DC)	< 0.11	0.18	nmol/mL	Serum	Short‐chain acylcarnitine
Ethanolamine	< 20	29	nmol/mL	Serum	Amino acid
Dodecanoylcarnitine (C12)	< 0.26	0.43	nmol/mL	Serum	Medium‐chain acylcarnitine
Dodecanedioylcarnitine (C12‐DC)	< 0.04	0.85	nmol/mL	Serum	Medium‐chain acylcarnitine
Cystathionine	< 4	13	nmol/mL	Serum	Amino acid
Orotic acid	0.4–1.2	1.4	mmol/mol Cr	Urine	Urine organic acid
AC/FC ratio	0.1–0.8	1.8	—	Serum	Ratio
Beta‐aminoisobutyric acid	< 5	7	nmol/mL	Serum	Amino acid
Aminobutyric acid	< 40	59	nmol/mL	Serum	Amino acid
Alpha‐aminoadipic acid	< 4	5	nmol/mL	Serum	Amino acid
Alanine	200–579	858	nmol/mL	Serum	Amino acid
3‐OH‐octadecanoylcarnitine (C18‐OH)	< 0.03	0.04	nmol/mL	Serum	Long‐chain acylcarnitine
1‐methylhistidine	< 20	12	nmol/mL	Serum	Amino acid

**Table 4:** Summary of Mayo Clinic Reference Laboratory biochemical genetics interpretive reports.

Test	Specimen	Key findings	Interpretation
Organic acid screen (Org AcdScrREF)	Urine	Elevated lactic acid and methylsuccinic acid; minimal dicarboxylic and 3‐hydroxy dicarboxylic aciduria; ethylmalonic acid, glutaric acid, and acylglycines not elevated	Supportive of riboflavin‐responsive multiple acyl‐CoA dehydrogenase deficiency (RR‐MADD), especially in context of the plasma acylcarnitine profile
Carnitine interpretation (Carnitn Int REF)	Plasma	Total and free carnitine reduced; relatively abundant esterified fraction; elevated AC/FC ratio (1.8)	Indicative of carnitine deficiency, possibly related to an underlying metabolic disorder including primary carnitine deficiency
Amino acid/quantitative profile (AAPQResultPREF)	Plasma	Multiple amino acids elevated; elevated peak most likely representing homocystine	In context of abnormal acylcarnitine profile, suggestive of riboflavin‐responsive MADD
Acylcarnitine comment (AcylcarnCommentREF)	Plasma	Multiple acylcarnitine species elevated across short‐, medium‐, and long‐chain lengths	Suggestive of ETF/ETF dehydrogenase deficiency (Glutaric acidemia type 2/MADD) or disorders of riboflavin metabolism

## 4. Discussion

This case highlights the importance of early consideration of metabolic disorders such as MADD in patients with unexplained metabolic derangements, particularly when initial workup for more common causes is negative. In the initial workup of this patient′s altered mental status, a differential diagnosis included infection, toxins including drug‐mediated effects from illicit substances or valproic acid, structural changes of the brain, and metabolic derangements. Illicit drug use was considered as the patient may have had exposure in prison, and synthetic tetrahydrocannabinol mimicking drugs such as K2 can present with encephalopathy and liver dysfunction leading to hyperammonemia [2]. Subsequent urine drug screens with cannabinoid testing were negative. Valproic acid and salicylate levels were negative. Imaging ruled out anatomic abnormalities of the brain.

In this case, the combined progressive laboratory abnormalities of elevated lactic acid, hyperammonemia, and hypoketotic hypoglycemia were inconsistent with expected manifestations of septic shock. Although there was radiographic evidence of hepatic steatosis, the patient′s normal transaminases, bilirubin levels, and normal PTT suggested that her elevated ammonia levels were disproportionate to her liver function at that time. Consideration of an underlying metabolic disorder was prompted after there was no significant change in encephalopathy with standard treatment of hyperammonemia and sepsis, including appropriate antibiotics, ammonia lowering agents, and renal replacement therapy.

Initial nonhepatic metabolic disorders considered included urea cycle disorders, mitochondrial diseases including fatty acid oxidation defects, organic acidemias, and dibasic amino acid deficiencies [3, 4]. Many of these are congenital inborn errors of metabolism. Multiple acyl‐CoA dehydrogenase deficiency is known, however, to present in females later in life [1, 4]. Other diseases with similar clinical manifestations and age of onset include medium chain or very long chain acyl coA dehydrogenase deficiency, carnitine aminotransferase 1a or II deficiency, glycogen storage diseases, primary carnitine deficiencies or carnitine acylcarnitine translocase deficiency [1]. Serum samples were sent to Mayo Clinic Reference Laboratory for acylcarnitine profiling (i.e., testing for fatty acid beta‐oxidation disorders and organic acidurias), as well as evaluation of carnitine and orotic acids. Urine studies would have provided more information but given the use of CRRT in this patient′s case, they were not obtainable.

In this case, results of the acylcarnitine testing interpreted by a biochemical geneticist demonstrated several distinctive features that together pointed toward a unifying diagnosis of MADD (Table 4). The hallmark elevated acylcarnitine species across all chain lengths suggest a disorder of ETF/ETF dehydrogenase seen in MADD [1]. These results have both overlying and distinct features from isolated fatty acid oxidation defects or secondary acylcarnitine derangements of critical illness in which typically specific chain length species, notably short chain acylcarnitine like acetylcarnitine (C2) species, are elevated [5]. Other supporting evidence of MADD includes elevated lactic acid and methylsuccinic acid on urine organic acids—a characteristic metabolite in MADD; as well as elevated sarcosine—the catabolism of which is ETF dependent and impaired in MADD; and elevated lysine—the catabolism of which is also impaired in MADD. Minimal dicarboxylic aciduria without elevated ethylmalonic acid or acylglycines is consistent with late‐onset MADD though is nonspecific.

A diagnosis of MADD can be sufficiently supported through identification of consistent acylcarnitine profiling with increased excretion of multiple organic acids in urine and/or by genetic identification of pathogenic variants in ETFA, ETFB, or ETFDH [1]. The patient′s rapid clinical deterioration limited additional confirmatory studies such as genetic testing, fibroblast in vitro probe analysis or temporal acylcarnitine testing [1]. These studies could have provided definitive evidence distinguishing the MADD presentation from critical illness related acylcarnitine abnormalities or alternative differential diagnoses. Early genetic testing—ideally initiated upon suspicion of MADD—would have informed more aggressive and personalized management.

The exact triggers of metabolic decompensation in MADD, particularly in this suspected case, warrant further exploration. Deficiencies such as MADD can manifest after catabolic stressors such as seizures, starvation, increased amounts of proteins through drugs or diet, or infections [1]. In this case, the patient experienced several days of nausea, vomiting, and decreased oral intake, which exacerbated her catabolic state, whereas pneumonia, urinary tract infection, the use of sedatives such as propofol, and isosource tube feedings were identified as possible contributors [1] (Table 2). It is important to discuss the mechanisms through which these factors exacerbate metabolic derangements: infection, in this case pneumonia, can lead to significant metabolic stress with increased demand for energy thus exacerbating fatty acid oxidation defects, which are central to MADD; Isosource contains medium chain fatty acids, and similarly, propofol contains long‐chain fatty acids. Both may overwhelm the already‐impaired beta‐oxidation pathway, further promote the accumulation of toxic intermediates, and worsen the patient′s metabolic state.

Early management of MADD is crucial for stabilization of mitochondrial function [6]. MADD management should include correction of hypoglycemia, bicarbonate therapy for reversal metabolic acidosis, and correction of carnitine deficiency with IV levocarnitine to prevent cardiac arrhythmias associated with accumulation of long‐chain acylcarnitine [1]. In this case, the carnitine panel revealed reduced total and free carnitine fractions with relatively abundant esterified fraction, which led to an elevated AC/FC ratio, suggestive of carnitine deficiency secondary to acylcarnitine formation. IV levocarnitine was started specifically because of the identified carnitine deficiency. The patient was monitored in the ICU with telemetry, ECG review, and electrolyte checks. Hyperammonemia should improve with reversal of catabolism—this can be medicated with high‐dose glucose infusion with insulin or in severe cases with extracorporeal toxin removal such as the renal replacement therapy provided in our patient′s case. Additionally, if rhabdomyolysis is present, hydration with 10% dextrose should be implemented to avoid catabolism associated with normal saline alone. Daily management also includes supplementation of CoQ_10_ to act as an electron acceptor in the ETF/ETFDH complex. High‐dose riboflavin can also help to stabilize the ETF/ETFDH complex [1, 7]. These measures were appropriately initiated in our patient, though potentially too late in her clinical course.

Though MADD is a diagnosis of exclusion due to its rarity, prompt identification of metabolic disorders in critically ill patients is essential for initiating targeted therapies. Given that MADD is a reversible condition in some cases, timely intervention might halt or slow the metabolic decompensation, reduce toxin burden, and reduce the chance of brain damage and death [3, 6, 8]. There are many case reports of late‐onset MADD, in which patients present mild symptoms [6, 9, 10]. This case highlights a fatal ICU presentation with diagnostic uncertainty, severe hyperammonemic encephalopathy, CRRT limiting urine diagnostic testing, and overlap with sepsis, shock, propofol exposure, and nutrition‐related lipid burden. Delayed diagnosis in complex ICU cases can impact patient prognosis and underscore the importance of metabolic screening in similar clinical presentations. A guide by Sulaiman et al. provides an algorithm for recognizing signs of metabolic decompensation such as hyperammonemia, severe metabolic acidosis and leucine encephalopathy—as well as correction measures for practitioners to employ [3].

In conclusion, clinicians should maintain a high index of suspicion for metabolic disorders in patients with altered mental status and otherwise unexplained metabolic derangements. Early recognition of these rare but serious conditions allows for timely intervention and may prevent fatal outcomes.

## Author Contributions

Drafting the manuscript: M.H., L.M., J.W., K.H., and N.M.Z. Evaluation of manuscript for content: K.H. and N.M.Z.

## Funding

No funding was received for this manuscript.

## Conflicts of Interest

The authors declare no conflicts of interest.

## Data Availability

The data that support the findings of this study are available from the corresponding author upon reasonable request.
